# Biodegradable Polymeric Microsphere-Based Drug Delivery for Inductive Browning of Fat

**DOI:** 10.3389/fendo.2015.00169

**Published:** 2015-11-09

**Authors:** Chunhui Jiang, Liangju Kuang, Madeline P. Merkel, Feng Yue, Mario Alberto Cano-Vega, Naagarajan Narayanan, Shihuan Kuang, Meng Deng

**Affiliations:** ^1^Department of Agricultural and Biological Engineering, Purdue University, West Lafayette, IN, USA; ^2^Bindley Bioscience Center, Purdue University, West Lafayette, IN, USA; ^3^College of Pharmacy, Purdue University, West Lafayette, IN, USA; ^4^Department of Animal Sciences, Purdue University, West Lafayette, IN, USA; ^5^Center for Cancer Research, Purdue University, West Lafayette, IN, USA; ^6^School of Materials Engineering, Purdue University, West Lafayette, IN, USA; ^7^Weldon School of Biomedical Engineering, Purdue University, West Lafayette, IN, USA

**Keywords:** Notch inhibition, browning, PLGA, microspheres, drug delivery, obesity

## Abstract

Brown and beige adipocytes are potent therapeutic agents to increase energy expenditure and reduce risks of obesity and its affiliated metabolic symptoms. One strategy to increase beige adipocyte content is through inhibition of the evolutionarily conserved Notch signaling pathway. However, systemic delivery of Notch inhibitors is associated with off-target effects and multiple dosages of application further faces technical and translational challenges. Here, we report the development of a biodegradable polymeric microsphere-based drug delivery system for sustained, local release of a Notch inhibitor, DBZ. The microsphere-based delivery system was fabricated and optimized using an emulsion/solvent evaporation technique to encapsulate DBZ into poly(lactide-*co*-glycolide) (PLGA), a commonly used biodegradable polymer for controlled drug release. Release studies revealed the ability of PLGA microspheres to release DBZ in a sustained manner. Co-culture of white adipocytes with and without DBZ-loaded PLGA microspheres demonstrated that the released DBZ retained its bioactivity, and effectively inhibited Notch and promoted browning of white adipocytes. Injection of these DBZ-loaded PLGA microspheres into mouse inguinal white adipose tissue depots resulted in browning *in vivo*. Our results provide the encouraging proof-of-principle evidence for the application of biodegradable polymers as a controlled release platform for delivery of browning factors, and pave the way for development of new translational therapeutic strategies for treatment of obesity.

## Introduction

The obesity epidemic has posed a major concern for public health in modern society due to its association with a spectrum of metabolic diseases, including type 2 diabetes (T2D), heart diseases, hyperglycemia, and multiple cancers (1, 2). Obesity is morphologically characterized by the excessive lipid storage in the white adipose tissue (WAT) and featured as a complex disorder of energy imbalance wherein intake outpaces expenditure. Considerable efforts have been devoted to explore the pathological mechanisms as well as the cellular and molecular therapeutic targets to combat obesity.

Currently, there are few medications available for obesity treatment (3). Mostly, those therapies are dedicated to decreasing energy intake by either suppressing appetite through stimulating the central nervous system or reducing nutrient digestion and absorption within the gastrointestinal tract (4). However, those medications only produce modest effects and are usually accompanied with unpleasant, potentially harmful side effects (5). Thus, alternative approaches to overcoming obesity are under extensive exploration. One such approach is through boosting energy expenditure. Recently, this strategy through stimulating thermogenesis has been emerging as an appealing alternative.

Brown adipose tissue (BAT) is the primary site of thermogenesis through uncoupling mitochondrial respiration and ATP synthesis via uncoupling protein 1 (Ucp1). While producing heat, BAT consumes not only free fatty acid but also large amounts of glucose (6, 7), thus providing benefits to metabolic health. Classical BAT is abundant in neonates but diminishes with age leading to limited quantities in adult humans. This compromises the therapeutic value of BAT in clinical intervention of obesity.

The recent discovery of inducible BAT (iBAT), also called beige adipose tissue, in WAT depots reignites the promise of obesity treatment through stimulating energy expenditure (8, 9). Beige adipocytes also possess robust thermogenic capacity, which has been confirmed recently by the transplantation of an engineered beige adipose tissue (10). More importantly, iBAT can be stimulated to burn energy in response to multiple pharmacological and genetic manipulations. Various factors have been identified to induce the transformation from white to beige adipose tissue (termed “browning”) over recent years (11, 12).

We have recently reported that inhibition of the Notch signaling pathway leads to browning of WAT, and thus ameliorates obesity (13). Notch signaling is well known to play fundamental roles in organ development and cell fate determination, but its regulatory roles in energy homeostasis has previously been underappreciated. We showed that genetic deletion of Notch1 receptor or the nuclear mediator of Notch signaling (Rbpj) in mice improved their glucose tolerance and insulin sensitivity through inducing browning of WAT. Importantly, Notch inhibition through intraperitoneal injection of dibenzazepine (DBZ), a γ-secretase inhibitor, similarly improved insulin sensitivity and glucose homeostasis. However, it remains elusive whether this systemic administration of DBZ causes any uncontrolled drug distribution and potential off-target side effects as Notch has been recognized to play multifaceted roles in various types of cells (14). Therefore, it is imperative to develop an effective drug delivery system to locally and continuously exert effects of Notch inhibition specifically on WAT.

Polymer-based drug delivery systems provide a robust technology platform to enable sustained, spatio-temporally controlled drug release (15). Biodegradable polymers are of utmost interest, because they can be broken down and excreted or resorbed by the body without the necessity for removal interventions or surgical procedures (16, 17). Biodegradable polymer microspheres with predetermined degradation and drug release profiles have been a primary research focus for development of effective drug delivery systems for a variety of applications, including cancer, cardiovascular diseases, and vaccine development (18, 19). Among a variety of polymers that have been employed for drug delivery, poly(lactide-*co*-glycolide) (PLGA), copolymers of lactic acid and glycolic acid, have been particularly prominent due to biocompatibility, biodegradability, and convenient processability (20). PLGA microspheres constitute a versatile class of drug delivery vehicles from which the drug release kinetics can be controlled by modulating the microsphere size, molecular weight, and composition of PLGA (21, 22).

**Figure 1:**
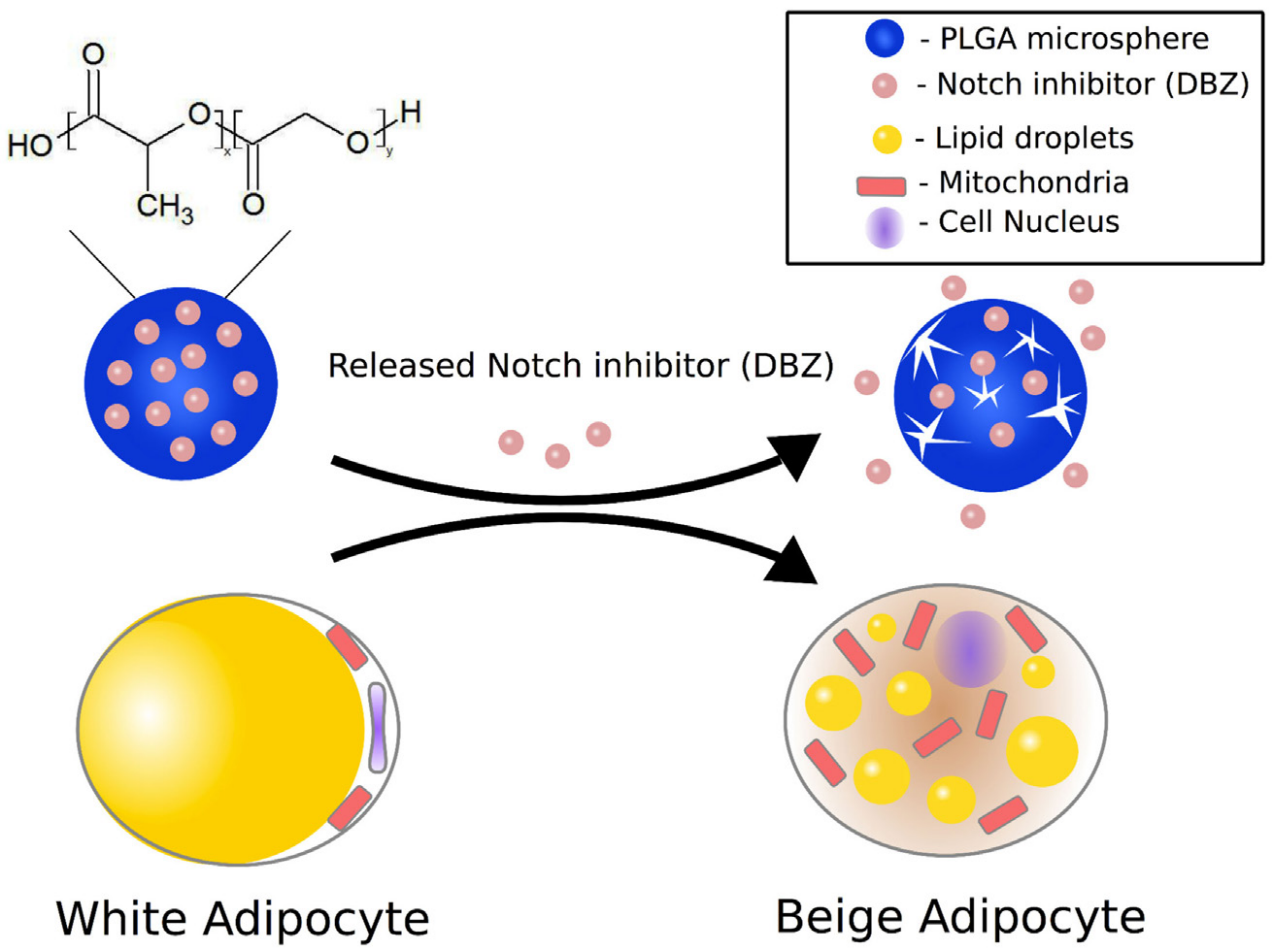
**Schematic illustration of DBZ-loaded PLGA microspheres enabling sustained release of DBZ to induce conversion of white adipocytes into beige adipocytes**.

In this study, we aimed to develop a PLGA microsphere-based DBZ delivery system to promote browning of WAT for the treatment of obesity (Figure 1). PLGA with a 50:50 lactide to glycolide ratio was utilized since this specific polymer has a relatively fast degradation rate among the PLGA family, which is advantageous to induce browning. We formulated the DBZ-loaded PLGA microspheres using an emulsion/solvent evaporation technique and characterized the morphology and release profiles of those microspheres. Moreover, we demonstrated that the DBZ-loaded PLGA microspheres inhibited Notch, and consequently promoted browning both *in vitro* and *in vivo*. To the best of our knowledge, this is the first time that an effective bioengineered system was developed to deliver therapeutic agents to convert white adipocytes to beige adipocytes. This study not only contributes to our understanding of the underlying mechanism of Notch inhibition in the browning process but also paves the way for development of novel therapeutic strategies to counteract obesity and its associated metabolic syndrome in humans.

## Materials and Methods

### Materials

PLGA (lactide:glycolide 50:50; Mw = 75,000) was obtained from lakeshore Biomaterials. Poly(vinyl alcohol) (PVA; 87–90% hydrolyzed, average Mw = 30,000–70,000), fluorescein isothiocyanate-labeled dextran (FITC-dextran) (Mw = 70,000), and DBZ were obtained from Sigma. Methylene chloride (DCM), dimethyl sulfoxide (DMSO), and acetonitrile were purchased from Fisher Scientific.

### Animals

All the mice used in this study were wild-type lean mice kept under normal maintenance in a clean facility at Purdue University. All procedures regarding animal maintenance and experimental use were conducted following the regulation presented by Purdue University’s Animal Care and Use Committee.

### FITC-Dextran-Loaded Microsphere Formulation and Characterization

Fluorescein isothiocyanate-labeled dextran-loaded PLGA microsphere was prepared by a water-in-oil-in-water (WOW) double emulsion technique (23). Briefly, 0.4 g of PLGA was dissolved in 4 mL DCM. Into this organic phase (O), 330 μL of aqueous solution (W1) containing ~3.7 mg of FITC-dextran was emulsified using a vortex mixer operating at 1,000 rpm for 3 min to form the W1/O emulsion. This primary emulsion was injected into 400 mL of an aqueous phase containing 1% (w/v) PVA (W2). The resulting W1/O/W2 emulsion was stirred at 400 rpm for overnight with an overhead magnetic stirrer to allow solvent evaporation and microspheres hardening. The microspheres were then isolated by centrifugation, washed three times with distilled water, and were dried in a vacuum oven for 24 h. Final products were stored in a desiccator. FITC-dextran-loaded PLGA microspheres were imaged by fluorescence microscopy (EVOS FL).

### DBZ-Loaded Microsphere Formulation and Characterization

PLGA microspheres loaded with DBZ were prepared by an oil-in-water (O/W) emulsion/solvent evaporation technique (24). The oil phase consisted of 540 mg of PLGA and 10.8 mg of DBZ dissolved in 2.7 mL DCM. This oil phase was injected into 400 mL of an aqueous phase containing 1% (w/v) PVA, which was stirred at 600 rpm to achieve an O/W emulsion system. The resulting emulsion was stirred overnight with an overhead magnetic stirrer to allow complete evaporation of the solvent and solidification of the droplets into microspheres. The microspheres were then isolated by centrifugation, washed three times with distilled water, and dried in a vacuum oven for 24 h. Final products were stored in a desiccator. The microsphere surface structure was investigated by scanning electron microscopy (SEM) (Nova Nano SEM). The microsphere diameter was measured using Image-J software, and about 130 microspheres were randomly selected for analysis.

The content of DBZ in microspheres was analyzed by a precipitation method. Five milligrams of DBZ-loaded PLGA microspheres were completely dissolved in chloroform. Once dissolved, the chloroform solution was added dropwise into 4 mL of methanol in a centrifuge tube to dissolve DBZ and precipitate PLGA. DBZ in the supernatant was collected and concentrated under nitrogen stream. Then, DBZ was dried in the vacuum oven for overnight and dissolved in 10 mL of 2:1 acetonitrile to Millipore water. The product was filtered for high-performance liquid chromatography (HPLC) analysis (Thermo HPLC). The samples were analyzed using a mobile phase of acetonitrile to 0.1% phosphoric acid (50:50) at a flow rate of 1 mL/min on a pentafluorophenylpropyl column and UV detection at 232 nm. Each sample was measured in duplicates. Actual drug loading and drug encapsulation efficiency were calculated using the following equations:

(1.1) $$\text{Theoretical DBZ loading}\;\%=\frac{\text{DBZ}(\text{Total})}{\text{DBZ}(\text{Total})+\text{PLGA}}\times 100\%$$

(1.2) $$\text{Actual DBZ loading}\;\%=\frac{\text{DBZ}(\text{Experiment})}{\text{DBZ}(\text{Total})+\text{PLGA}}\times 100\%$$

(1.3) $$\text{Encapsulation efficiency}\;\%=\frac{\text{Actual DBZ loading}}{\text{Theoretical DBZ loading}}\times 100\%$$

### *In vitro* FITC-Dextran Release from Microspheres

Fluorescein isothiocyanate-labeled dextran released from microspheres was measured by suspending ~20 mg microspheres in 5 mL PBS buffer at pH 7.4. The samples were placed in a shaker maintained at 37°C and shaken at 200 rpm. At predetermined time intervals, the samples were removed from the shaker and centrifuged at 1,000 × *g* for 2 min. Two milliliters of medium was aliquoted for analysis and fresh medium of equal volume was added thereafter. The precipitated microsphere pellets were resuspended in the medium and placed back in the shaker. *In vitro* release of FITC-dextran was studied in triplicate. The FITC-dextran concentration in the aqueous phase was determined fluorometrically (excitation: 485 nm, emission: 520 nm, Synergy H1 microplate reader) using a standard calibration curve.

### *In vitro* DBZ Release from Microspheres

The DBZ-loaded PLGA microspheres were characterized for drug release for 6 days in PBS at pH 7.4 and 37°C. DBZ release was measured using HPLC. In brief, 20 mg samples were placed in individual centrifuge tubes and filled with 2.5 mL of PBS. The samples were then placed in a shaker maintained at 37°C and shaken at 200 rpm. At specific time points, 1 mL medium was removed, saved for analysis, and replaced with same amount of fresh PBS. Time points were chosen such that perfect sink conditions were maintained. Each supernatant sample was extracted with 1 mL chloroform. The organic layer was then separated and allowed to evaporate. The dried DBZ was then reconstituted with 1 mL of a 40% solution of acetonitrile in water to provide a suitable solution for HPLC analysis (Thermo HPLC).

### Isolation of Primary Preadipocytes

Primary preadipocytes were collected from limb subcutaneous WAT depots and minced into 2–5 mm^2^ pieces. Then, these pieces were subject to 1.5 mg/mL collagenase digestion with agitation at 37°C for 1.5–2 h. The digestion was terminated with Dulbecco’s modified Eagle’s medium (DMEM) containing 10% fetal bovine serum (FBS). After that, the floating mature adipocytes were removed and the cell suspension was filtered through 100 μm mesh, followed by brief centrifugation at 450 × *g* for 5 min. The pellet was resuspended and seeded onto tissue culture plates.

### Co-Culture of Primary Preadipocytes with Microspheres

Primary preadipocytes were cultured in growth medium containing DMEM, 20% FBS, and 1% penicillin/streptomycin at 37°C with 5% CO_2_. The medium was changed every other day. Upon confluence, cells were subject to induction medium containing DMEM, 10% FBS, 2.85 μM insulin, 0.3 μM dexamethasone, and 0.63 mM 3-isobutyl-methylxanthine for 4 days, followed by differentiation medium containing DMEM, 200 nM insulin, and 10 nM triiodothyronine for four more days. Meanwhile, 5 mg polymer microspheres were added in permeable transwell inserts suspended in each well of 24-well plates while cells were induced for adipogenic differentiation. Other parallel treatment groups included cells in DBZ-containing medium (10 μM) and cells in DMSO vehicle control. To monitor adipogenic differentiation, the lipid droplets and nuclei of adipocytes during culture were counterstained with BODIPY and DAPI, respectively.

### Quantitative Polymerase Chain Reaction

Total RNA was extracted from cell culture through Trizol. Random hexamer primers were utilized for the reverse transcription to synthesize cDNA. The quantitative polymerase chain reaction (qPCR) was performed with a Roche Light Cycler 96 machine (Roche). The 18S rRNA was applied as an internal control for normalization. For qPCR result analysis, the 2^−ΔΔct^ method was used to calculate the fold change.

### Protein Extraction and Western Blots Analysis

Total protein was extracted from cells or tissue samples using RIPA buffer containing 50 mM Tris–HCl (pH 8.0), 150 mM NaCl, 1% Non-idet P-40, 0.5% sodium deoxycholate, and 0.1% sodium dodecyl sulfate (SDS). Protein concentrations were measured by using Pierce BCA protein assay reagent (Pierce Biotechnology, Rockford, IL, USA). Proteins were separated by SDS-polyacrylamide gel electrophoresis (SDS-PAGE) and then transferred to polyvinylidene fluoride (PVDF) membranes (Millipore Corp., Billerica, MA, USA). Membranes were blocked in 5% milk for 1 h and incubated with primary antibodies at 4°C overnight. The PGC1-α (sc-13067) and GAPDH (sc-32233) antibodies were purchased from Santa Cruz Biotechnology, and both were diluted 1:1,000. The horseradish peroxidase (HRP)-conjugated secondary antibody (anti-rabbit IgG, 7074S, Cell Signaling) was diluted 1:5,000. Signals were detected with a ChemiDoc™ Touch Imaging System (Bio-Rad). The lanes were analyzed for densitometry quantification with Bio-Rad Image Lab V5.2.1.

### *In vivo* Injection of PLGA Microspheres

Mice were first anesthetized by a ketamine–xylazine cocktail and then either FITC-dextran-loaded PLGA microspheres or DBZ-loaded PLGA microspheres (20 mg/30 g body weight) were injected into the inguinal WAT depot in one side of the body in a 1 mL solution of 0.5% Methocel E4M (wt/vol; Dow Chemical) and 0.1% Tween-80 (wt/vol; Sigma) in water. Similarly, PLGA microspheres were injected into the contralateral inguinal WAT depot. Adipose tissues were harvested after 24 h and 14 days post-injection for the study with FITC-dextran-loaded PLGA microspheres and DBZ-loaded PLGA microspheres, respectively. Oil Red O staining was performed to label the adipocytes for the study with FITC-dextran-loaded PLGA microspheres whereas hematoxylin and eosin (H&E) and immunohistochemistry staining was conducted for the study with DBZ-loaded PLGA microspheres.

### H&E and Immunohistochemistry Staining

Adipose tissues were fixed in 10% formalin for 24 h at room temperature. Then, the tissues were embedded into paraffin and cut into 4 μm thick slices, deparaffinized, and rehydrated using xylene, ethanol, and water by standard methods. Immunohistochemistry was performed on a Dako Autostainer (Dako). Slides were incubated with 3% hydrogen peroxide and 2.5% normal horse serum (S-2012, Vector), followed by incubation with rabbit polyclonal anti-Ucp1 primary antibody diluted 1:200 in 2.5% normal horse serum (S-2012, Vector) for 60 min. Signals were detected with an anti-rabbit IgG Polymer Detection Kit (MP-7401, Vector). Labeling was visualized with 3,3′-diaminobenzidine (DAB) as the chromogen (SK-4105, Vector). Slides were counterstained with Harris hematoxylin (EK Industries), and whole-slide digital images were collected with an Aperio Scan Scope slide scanner (Aperio).

### Statistical Analysis

Quantitative data were reported as mean ± SEM *P*-values were calculated by a two-tailed Student’s *t*-test. *P* < 0.05 was considered to be statistically significant.

## Results

### Formulation and Characterization of PLGA Microspheres

**Figure 2:**
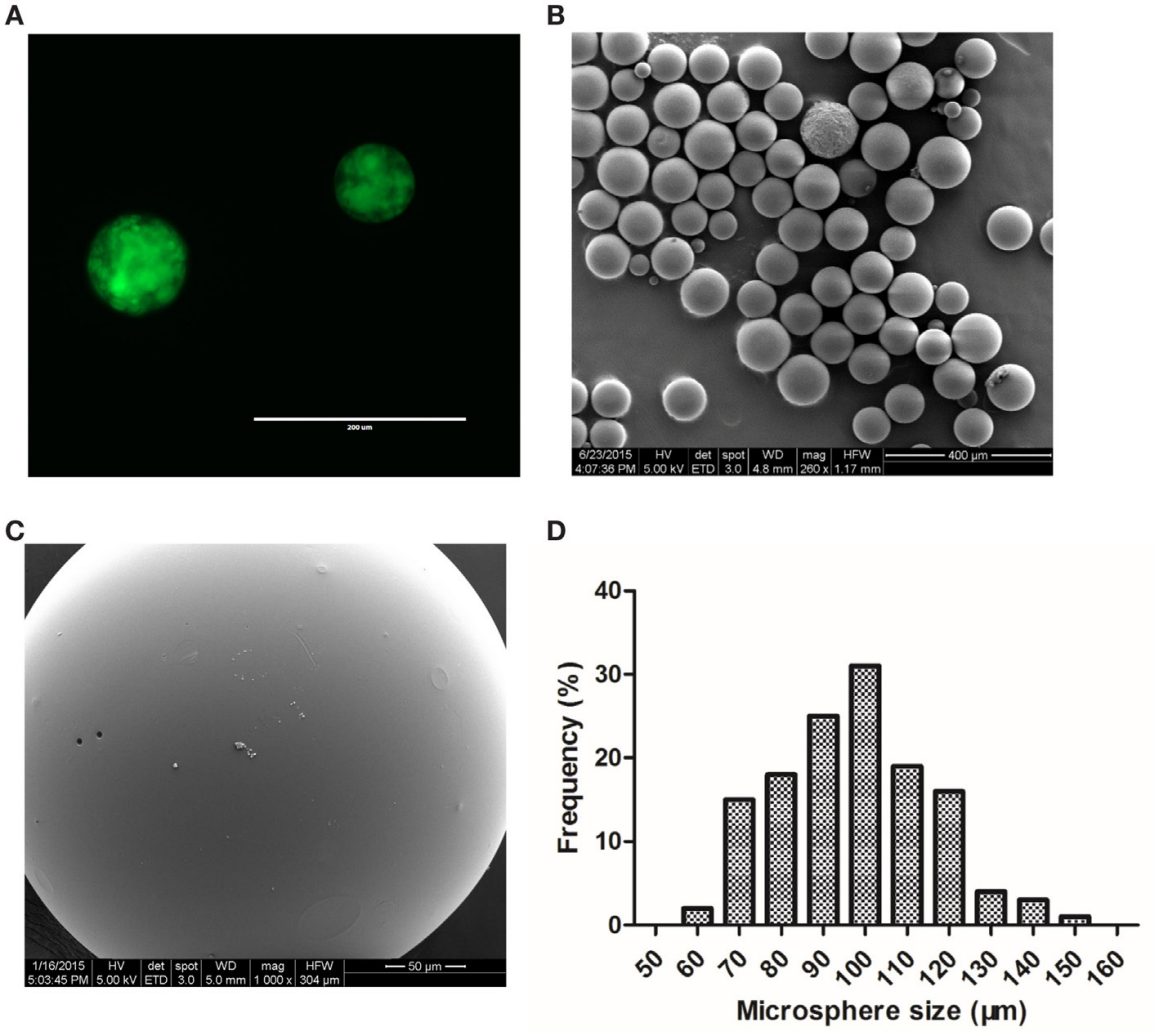
**Characterization of FITC-dextran and DBZ-loaded PLGA microspheres**. **(A)** Fluorescent image of FITC-dextran-loaded microsphere. Scale bar, 200 μm. **(B)** SEM image of a population of DBZ-loaded PLGA microspheres. Scale bar, 400 μm. **(C)** Higher magnification SEM image of a single DBZ-loaded PLGA microsphere. Scale bar, 50 μm. **(D)** Size distribution of DBZ-loaded PLGA microspheres.

Using PLGA 50:50, drug-loaded PLGA microspheres were prepared and characterized for drug distribution and morphological characteristics. We first encapsulated FITC-dextran, a commonly used fluorescent probe, into PLGA microspheres to assess the distribution of FITC-dextran in microspheres by fluorescent microscopy. It was observed that green fluorescence was uniformly distributed throughout the microspheres (Figure 2A). Next, we formulated DBZ-loaded PLGA microspheres by optimizing the process parameters, such as PVA concentration and stirring speed, to obtain a narrow microsphere size range of 50–150 μm (Figure 2B). Higher magnification SEM micrograph revealed the smooth surface morphology of the microsphere (Figure 2C). The size distribution of the DBZ-loaded PLGA microspheres resembled a Gaussian distribution with more than 50% in the range of 90–110 μm (Figure 2D). The actual DBZ loading percentage was characterized to be ~1.4% (w/w), equivalent to ~68% DBZ encapsulation efficiency.

### PLGA Microsphere System Enables FITC-Dextran and DBZ Release

**Figure 3:**
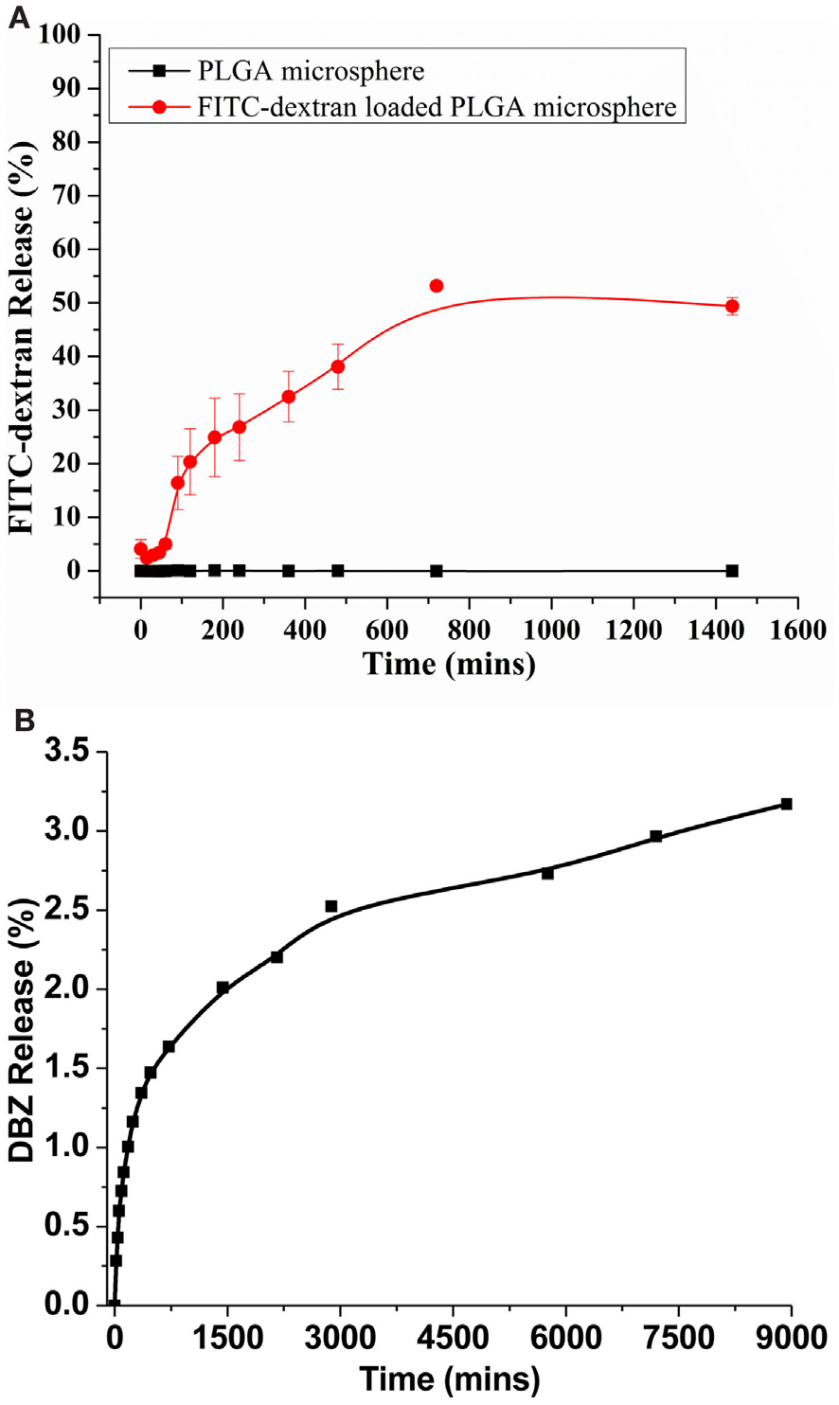
***In vitro* release profiles of FITC-dextran and DBZ from PLGA microspheres**. **(A)** *In vitro* release of FITC-dextran from microspheres in PBS at pH 7.4 and 37°C. **(B)** *In vitro* release of DBZ from microspheres in PBS at pH 7.4 and 37°C.

To examine the cargo drug release capabilities from the PLGA microspheres, we first placed the FITC-dextran-loaded PLGA microspheres in the PBS at 37°C and release was quantified fluorometrically. The FITC-dextran-loaded PLGA microspheres showed a rapid release profile, which resulted in about 50% of total FITC-dextran released after 24 h (Figure 3A). This is presumably due to the hydrophilic nature of the FITC-dextran. We further characterized the release profile of DBZ from DBZ-loaded PLGA microspheres (Figure 3B). In general, the release rate of DBZ from PLGA microspheres was much slower than that of FITC-dextran. Approximately, 2% DBZ was released from PLGA microspheres in the first 24 h. And the release rate became significantly decreased thereafter and around 3% DBZ (~8 μg) was released over 6 days, indicating that DBZ can be released from PLGA in a sustained manner.

### DBZ-Loaded PLGA Microspheres Promote Browning in Adipocytes

**Figure 4:**
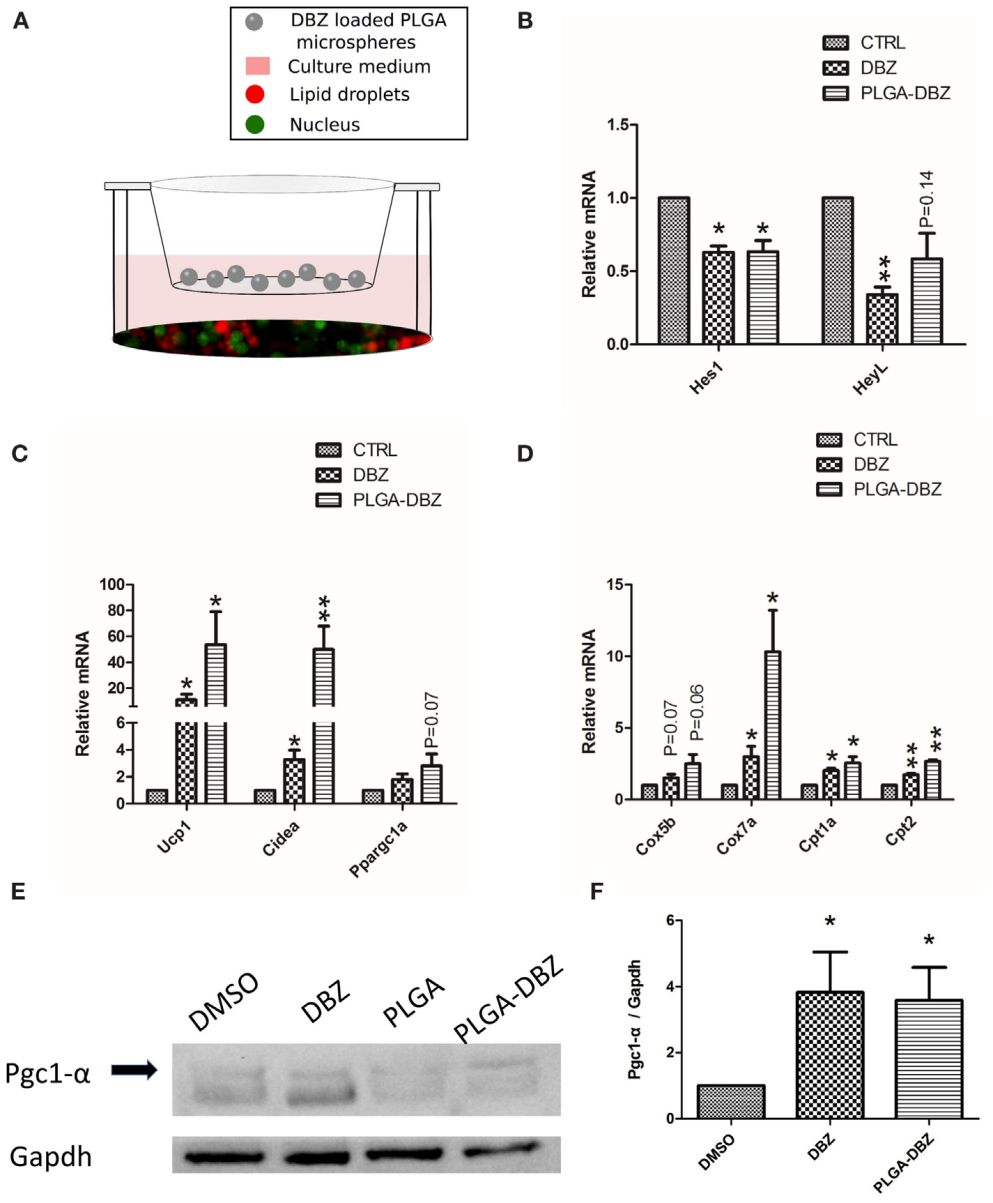
**DBZ-loaded PLGA microspheres inhibit Notch and promote browning *in vitro***. **(A)** Diagram of the co-culture system with primary white adipocytes seeded on well bottom and DBZ-loaded microspheres placed within a permeable insert. Mature adipocytes were labeled with BODIPY (red). Nuclei were counterstained with DAPI (green). Expression of **(B)** Notch target genes, **(C)** browning markers, and **(D)** mitochondria genes in white adipocytes co-cultured with DBZ-loaded PLGA microspheres. **(E)** Protein levels of Pgc1-α in white adipocytes co-cultured with DBZ-loaded PLGA microspheres. **(F)** Quantification of protein level for Pgc1-α relative to Gapdh. DBZ: cells in DBZ-containing medium (10 μM); CTRL: cells treated with DMSO vehicle control. *N* = 3. *P < 0.05, **P < 0.01.

As the initial attempt to test the bioactivity of DBZ released from PLGA microspheres, we co-cultured DBZ-loaded PLGA microspheres with primary adipocytes and examined the cellular responses (Figure 4A). Following induction of adipogenic differentiation for 8 days, we observed classical lipid droplets characterized by the BODIPY immunofluorescence (Figure 4A), which suggested that microspheres did not cause obvious adverse effects on cell culture. Then, we continued to analyze the mRNA levels of Notch target and thermogenic genes. As we expected, DBZ-loaded PLGA microspheres significantly inhibited Notch, demonstrated by the 40% mRNA reduction of Notch target genes, *Hes1* and *HeyL* (Figure 4B). Importantly, DBZ-loaded PLGA microspheres dramatically increased mRNA levels of browning markers, including *Ucp1*, *Cidea*, and *Ppargc1a* (Figure 4C), as well as mitochondria genes, including *Cox5b*, *Cox7a*, *Cpt1a*, and *Cpt2* (Figure 4D), suggesting that DBZ released from PLGA microspheres can inhibit the Notch pathway and consequently promote browning. In addition, western blot results shown in Figure 4E demonstrated that DBZ-loaded PLGA microspheres significantly elevated the protein level of Pgc1-α (encoded by *Ppargc1a*) by around 3.5-fold (Figure 4F), which plays key roles in mitochondrial biogenesis and oxidative metabolism. Collectively, all these data confirmed that DBZ remained bioactive to induce browning after being released from PLGA microspheres *in vitro*.

### DBZ-Loaded PLGA Microspheres Stimulate Browning *In vivo*

**Figure 5:**
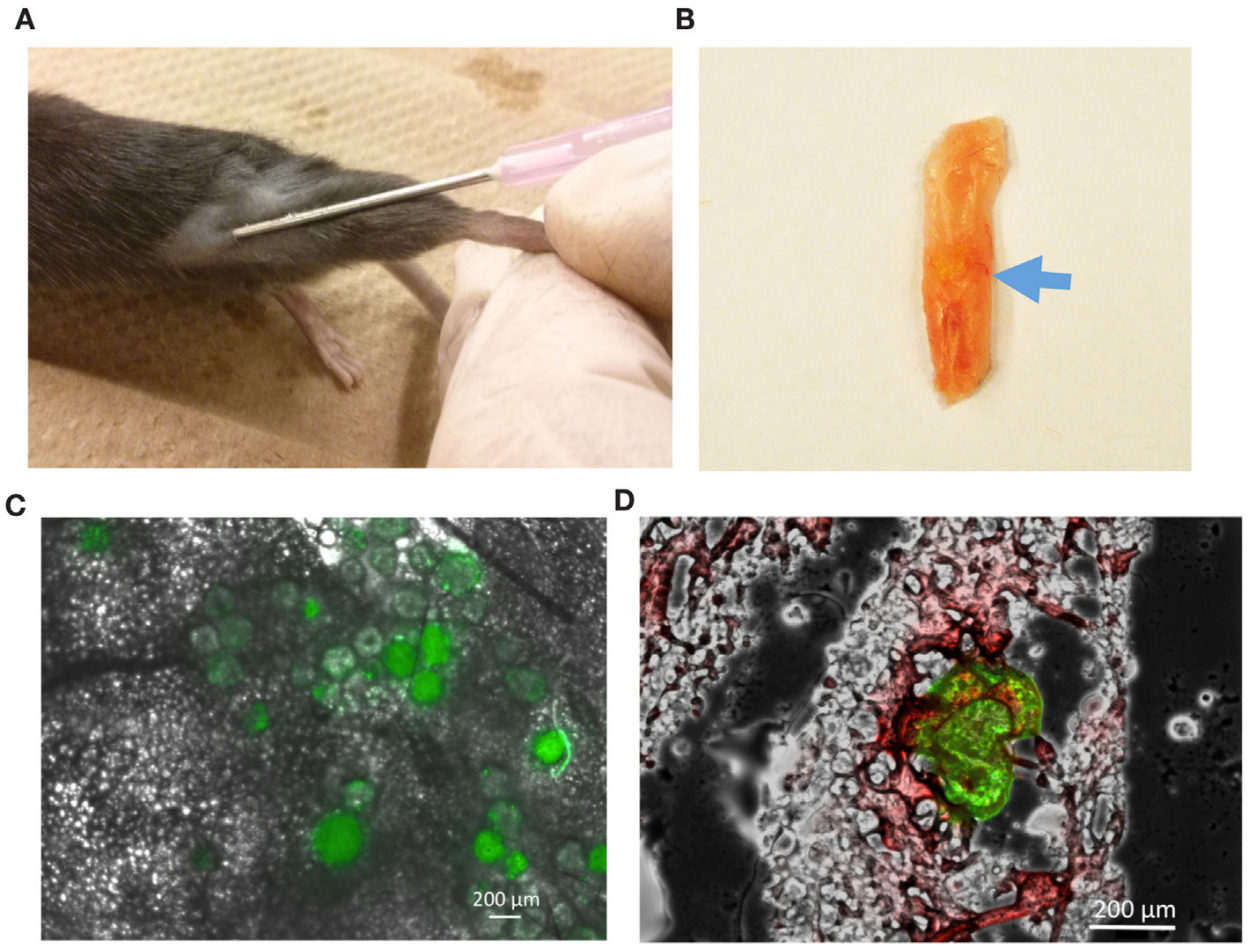
**Characterization of FITC-dextran-loaded microspheres following injection into mice**. **(A)** Direct injection of microspheres into inguinal adipose depot of WT mice. **(B)** Injected microspheres (blue arrow) identified through gross observation of inguinal adipose tissues. **(C)** Fluorescent image illustrating the distribution of microspheres after 24 h of injection. **(D)** Representative image showing green fluorescence from FITC-dextran-loaded microspheres dispersed in white adipocytes labeled by Oil Red O staining. Green: FITC; Red: Oil Red O.

To further test whether PLGA microspheres can locally deliver DBZ and promote browning *in vivo*, we sought to directly inject microspheres to the mouse inguinal WAT depots and assess the browning effect thereafter (Figure 5A). Initially, to ensure that microspheres could be precisely injected into the depots, we utilized the FITC-dextran-loaded microspheres to track microsphere placement. The microsphere injection sites were readily identified with green fluorescence via gross observation in the target inguinal adipose tissues (Figure 5B). Following 24 h of injection, FITC-dextran-loaded microspheres maintained round morphologies with expanded fluorescence, indicating the release of FITC-dextran from the microspheres over this time period (Figures 5C,D). This observation was also in agreement with our previous results regarding the release profile of FITC-dextran *in vitro*.

**Figure 6:**
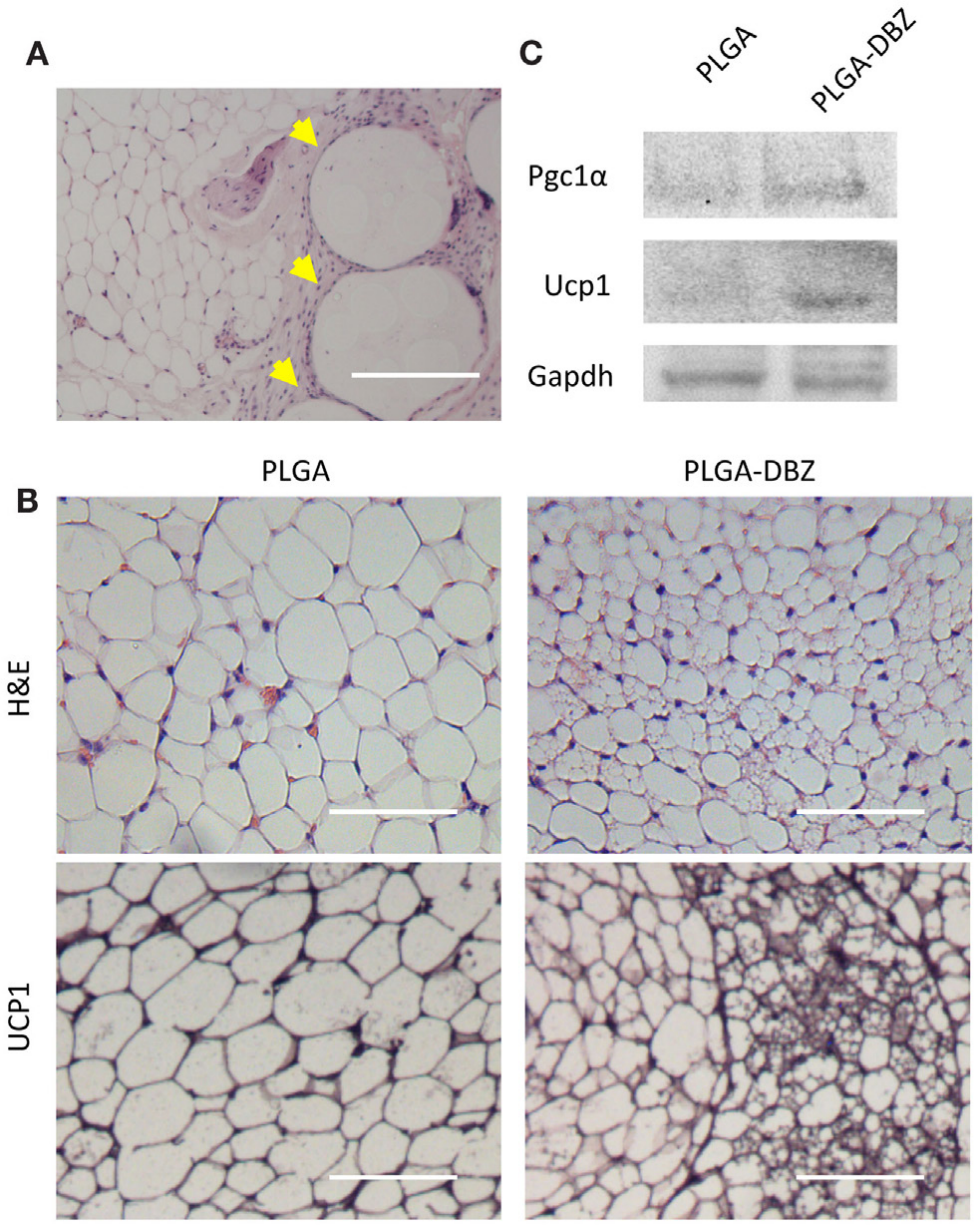
**DBZ-loaded PLGA microspheres induce browning *in vivo***. **(A)** H&E staining showing DBZ-loaded PLGA microspheres (yellow arrowhead) dispersed within the WAT depot. Scale bar, 200 μm. **(B)** H&E (top) and UCP1 (bottom) staining of inguinal WAT from mice at 14 days post-injection of DBZ-loaded PLGA microspheres. Scale bars, 100 μm. **(C)** Protein levels of Pgc1-α and Ucp1 of inguinal WAT from mice at 14 days post-injection of DBZ-loaded PLGA microspheres.

We next injected DBZ-loaded PLGA microspheres into the inguinal WAT depots using the same procedure. Fourteen days after injection, the inguinal WAT was collected and processed for paraffin embedding and sectioning. Through H&E staining, we observed that DBZ-loaded PLGA microspheres were dispersed within the WAT and maintained round morphologies in the size range of 50–150 μm. Importantly, these microspheres did not elicit any significant inflammatory responses (Figure 6A). Of note, DBZ-loaded PLGA microspheres drastically decreased the sizes of mature white adipocytes (Figure 6B), a hallmark indicator of browning effect. Multilocular Ucp1^+^ beige adipocytes were evidently abundant with DBZ-loaded PLGA microspheres (Figure 6B). Also, this morphological feature of browning was further supported by western blot of tissue samples exhibiting increased protein levels of Pgc1-α and Ucp1 in the DBZ-loaded microsphere group compared to control (Figure 6C). All these data demonstrated that DBZ was successfully released from microspheres and retained its biological activity of Notch inhibition to promote browning *in vivo*.

## Discussion

There is accumulating evidence that significant metabolic differences that distinguish beige adipocytes from white adipocytes can potentially be successfully exploited to establish new therapeutic strategies for treatment and/or prevention of obesity. Thus, identifying and targeting mechanisms underlying browning is crucial in the development of effective therapeutics to reduce adiposity. The Notch signaling pathway is important for cell–cell communication and cell fate determination during development and is required for adult tissue homeostasis. Notch target gene Hairy/enhancer-of-split 1 (Hes1) inhibits the transcriptions of PR domain-containing 16 (Prdm16), peroxisome proliferator-activated receptor gamma (Pparγ) coactivator 1 alpha (Ppargc1a) and Pparγ, all critical in the brown adipocyte biogenesis (13). This leads to reduced mitochondrion numbers and expression of Ucp1 (25–27). Notably, inhibition of Notch signaling through intraperitoneal injection of DBZ has been found to induce browning of white adipocytes, and consequently reduced obesity and improved glucose balance in obese mice (13). This strategy has been proposed to open up another novel avenue to treat obesity and its associated metabolic diseases. However, potential compliance issues associated with a requirement for multiple periodic drug injections, in combination with safety and efficacy concerns over widespread drug distribution in the body reduce the translational potential of this treatment. For instance, Notch inhibition has been proposed to affect multiple biological processes, such as angiogenesis (28, 29), bone formation (30, 31), and myogenesis (32, 33). Therefore, it would be beneficial if a carrier system were capable of delivering DBZ locally to the WAT in a sustained manner for treatment of obesity.

Polymer-based controlled drug delivery systems offer several unique advantages over conventional drug delivery including continuous drug release, decreased systemic side effects, and increased patient compliance (15). Polymer degradation is highly desirable in controlled drug delivery because it eliminates the need for the surgical removal of implants. PLGA is known to degrade by simple hydrolysis of the ester bonds into lactic and glycolic acids, which are ultimately metabolized to carbon dioxide and water (34). PLGA microspheres have been extensively investigated as carriers to deliver a variety of therapeutic agents (21, 22). The release of an encapsulated agent from PLGA microspheres is controlled by both diffusion and polymer degradation. The drug release kinetics can be tailored by varying the copolymer composition, molecular weight, and microsphere size (20–22). In particular, the degradation rate of PLGA depends on the copolymer composition. The molecular weight loss during hydrolysis is accelerated with an increase in glycolide content. This is attributed to greater absorption of water into the polymer matrix. For example, PLGA exhibited a degradation time in the order of PLGA 50:50 (1–2 months) < PLGA 75:25 (4–5 months) < PLGA 85:15 (5–6 months) (35). Polymers with higher molecular weight result in slower degradation rates (36). Furthermore, the size of PLGA microspheres is an important controlling factor on drug diffusion and polymer degradation. Berchane et al. demonstrated that the initial burst release rate of piroxicam from PLGA microspheres decreased with an increase in microsphere size from 13.9 to 81.2 μm, which is consistent with Fick’s law of diffusion because an increase of diffusion pathways would reduce the drug release rate (37). It has been reported that large microspheres degrade in a heterogeneous manner wherein the degradation rate in the core is greater than that at the surface (38). In contrast, microspheres with diameter <300 μm undergo a homogeneous degradation whereby the core degradation rate is equivalent to the surface one (39). Small microspheres also degrade slower than large microspheres resulting from a decreased accumulation of acidic degradation products (40). *In vitro* release studies showed that the fabricated DBZ-loaded PLGA microspheres in the size range of 50–150 μm enabled sustained release of DBZ resulting in ~3% of encapsulated drug released over 6 days. This gradual release will be particularly beneficial to circumvent the need for multiple periodic injections of DBZ for induction of browning.

Co-culture of primary preadipocytes with DBZ-loaded PLGA microspheres demonstrated that released DBZ retained its bioactivity and effectively inhibited Notch signaling and upregulated expression of brown fat specific genes (Figure 4). For example, the expression of Notch downstream targets *Hes1* and *HeyL* in cultured white adipocytes with both DBZ and DBZ-loaded microspheres were reduced by more than 40%, indicating the bioactivity of DBZ released from polymers. Furthermore, DBZ-loaded microspheres resulted in an elevated expression of browning markers, including *Ucp1*, *Cidea*, and *Ppargc1a*, as well as mitochondria genes, including *Cox5b*, *Cox7a*, *Cpt1a*, and *Cpt2*. Notably, brown and beige adipocytes contain more mitochondria than white adipocytes, and possess ability to burn lipids (through β-oxidation) to generate heat (11). Pgc1-α (encoded by *Ppargc1a*) is also known to induce the expression of Ucp1 and other thermogenic components in adipocytes (26, 41). Our results are also in line with our earlier findings with the use of DBZ on browning of white adipocytes (13). The more pronounced increase in *Ucp1* and *Cidea* mRNA levels in the PLGA-DBZ-treated groups compared to the DBZ alone groups is presumably due to the continued release of DBZ from microspheres, which should have improved the biological activity of DBZ and thus highlights a major advantage of our drug delivery system for inductive browning.

One of the most important requirements for developing effective polymer-based drug delivery systems is tissue compatibility. PLGA polymers are considered a standard for drug delivery due to their recognized biocompatibility and approval by the Food and Drug Administration for a number of clinical applications (16, 17). The DBZ-loaded PLGA microspheres were well tolerated following *in vivo* injection into the WAT depots (Figure 6A). More importantly, we demonstrated that local DBZ-loaded microsphere injection successfully induced browning of WAT after 14 days (Figures 6B,C). The resultant browning further validates that Notch signaling plays a role in regulating the plasticity of white and beige adipocytes *in vivo* (13).

White adipose tissue is the primary site of long-term energy storage. In response to excess calorie intake, the size of the WAT expands through hyperplasia and hypertrophy of adipocytes. Our work pinpointed the potential of polymeric microspheres as a carrier platform to locally deliver DBZ to WAT in a sustained manner. Such a localized delivery system may eliminate the need for repeated injections as well as potential side effects that are accompanied with systemic administration of DBZ. Improved understanding of brown and beige adipocyte biology has led to recent discoveries of a variety of factors to promote WAT browning, such as fibroblast growth factors (42–44), bone morphogenetic proteins (BMPs) (45, 46), Irisin (47), T3 and T4 thyroid hormones (45, 46), natriuretic peptides (48), and β3-adrenergic pathway agonist (49–51). However, as with DBZ, there exist significant safety and efficacy concerns on systemic administration of these browning factors as uncontrolled tissue exposure may lead to potential off-target side effects and unpredictable kinetics. For instance, administration of FGF21 is associated with systemic side effects on bone loss (52). Therefore, development of effective browning-based therapeutics to address the ever-worsening obesity epidemic necessitates the integration of advances in controlled release technologies with discoveries in beige/brown fat cell biology.

## Conclusion

DBZ-loaded PLGA microspheres with a size range of 50–150 μm were prepared as a DBZ delivery system using an emulsion/solvent evaporation technique. The DBZ-loaded PLGA microspheres supported release of DBZ in a sustained manner. The effectiveness of DBZ-loaded PLGA microspheres to induce browning was demonstrated by both *in vitro* and *in vivo* studies. This study for the first time demonstrates the feasibility of developing a bioengineered carrier system for controlled delivery of DBZ to achieve inductive browning using biodegradable polymeric microspheres. To facilitate the translation of our technology platform to have a positive impact in the therapeutic treatment, future efforts will be focused on the evaluation of this controlled delivery system with human cells and tissues.

## Authors Contribution

MD, SK, CJ, and LK conceived and designed experiments. CJ, LK, MM, FY, MC-V, and NN performed experiments. CJ, LK, and MD analyzed the data. CJ, LK, MM, MD, and SK wrote the manuscript. MD had full access to all the data of the study and takes responsibility for the integrity of the data and accuracy of data analysis.

## Conflict of Interest Statement

The authors declare that the research was conducted in the absence of any commercial or financial relationships that could be construed as a potential conflict of interest.
